# Fabrication and Test of an Inflated Circular Diaphragm Dielectric Elastomer Generator Based on PDMS Rubber Composite

**DOI:** 10.3390/polym9070283

**Published:** 2017-07-15

**Authors:** Giacomo Moretti, Michele Righi, Rocco Vertechy, Marco Fontana

**Affiliations:** 1 TeCIP Institute, Scuola Superiore Sant’Anna, P.zza dei Martiri, 33, 56127 Pisa, Italy; g.moretti@santannapisa.it (G.M.); mi.righi@santannapisa.it (M.R.); 2 Department of Industrial Engineering, University of Bologna, Viale del Risorgimento, 2, 40136 Bologna, Italy; rocco.vertechy@unibo.it; 3 Department of Industrial Engineering, University of Trento, Via Somarive, 9, 38123 Trento, Italy

**Keywords:** dielectric elastomer, PDMS devices, variable capacitance, electrostatic generators

## Abstract

This paper introduces a fabrication method and the experimental characterization of a soft polymeric energy converter manufactured using a combination of dielectric and conductive polydimethylsiloxane elastomers. The presented system is an inflated circular diaphragm dielectric elastomer generator; i.e., a deformable electrostatic transducer that converts the mechanical work done by a time-varying pressure into electricity. A prototype of the system is realized on the basis of a simple fabrication procedure that makes use of commercially available silicone dielectric elastomer films and custom-prepared deformable conductive electrodes. A test-bench is developed and employed to estimate the energy conversion performance. Remarkable results are obtained, such as an amount of energy converted per cycle of up to 0.3 J, converted power of up to 0.15 W, energy per unit of employed elastomer mass of up to 173 J/kg, and fraction of the input mechanical work converted into electricity of 30%.

## 1. Introduction

Energy harvesting is a central application area for multifunctional materials. In the attempt to achieve low-cost direct-drive electricity generation from ambient sources of mechanical energy, different technologies have indeed been investigated, which include piezoelectric [1], triboelectric [2], and electrostatic [3] generators. A special class of electrostatic generators based on polymeric materials are the dielectric elastomer transducers (DETs). DETs are soft/deformable elastomeric capacitors comprising one or more layers of a dielectric elastomer (DE) film coated by compliant electrodes. They can be employed as generators (DEGs) to transform an input mechanical energy into electricity, or as actuators (DEAs) to produce forces and displacements regulated by electrical activation. DEGs were first introduced by SRI, and since then, they have attracted a great deal of interest for their promising performance in applications such as ocean wave energy converters (WECs) and portable/wearable energy harvesters [4]. Several DEG architectures and layouts have been studied, including devices where the capacitive membranes are shaped as cones, lozenges, or stacks, subjected to mono-axial, multiaxial, or equibiaxial states of stretch [5,6,7].

To date, the DE materials employed for DEG demonstrators and prototypes have mostly been polyacrylates and natural rubber [8,9]. The use of such materials has proven the capability of the DEG technology to deliver high convertible power and energy densities, comparable to those of the other above-mentioned energy harvesting technologies. This has been possible thanks to their ability to sustain large stretches and high electric fields, which are further enhanced during cyclical loading [10,11,12,13], combined with a low mass density.

From the perspective of industrialization and commercialization, however, it seems that the most suitable elastomers for DEG application are polydimethylsiloxane (PDMS) silicone elastomers [14]. Compared to other DE materials, PDMS elastomers potentially enable highly repeatable manufacturing processes, which yield high-quality elastomeric matrices free of flaws and inclusions, and show little tendency to stress relaxation, low viscosity, and good long-term stability [15]. Moreover, silicone polymers provide good potential for chemical modifications, which in the last years has led to several attempts to enhance the electro-mechanical properties of available materials by acting on their macromolecular structure [16,17,18].

Besides the active dielectric material, a crucial component of DE transducers is represented by the compliant electrodes, which are required to provide high extensibility and to be electrically conductive. It has been suggested that PDMS-based DE transducers might be equipped with compliant electrodes made of carbon-doped PDMS layers that can be bonded on the silicone DE substrate to which they are chemically affine, so as to provide a compact monolithic DE transducer assembly [19].

PDMS as DE material has been almost exclusively employed to build DE actuators [15,20], whereas only a small number of works on energy harvesting with PDMS-based DEGs is currently available. Specifically, a miniature stack-generator has been designed and built [7] showing suitable performance in terms of power conversion capabilities for small-scale energy harvesting. In addition, PDMS-DEGs that operate close to mono-axial stretching conditions have been studied [21,22]. Besides the evaluation of absolute power capabilities, those experimental tests reported achieved energy/power densities (per unit of active DE mass) in the range of units of joules/watts per kilogram (i.e., two orders of magnitude lower than DEGs made of natural rubber or acrylics) [8,9,23], while no information has been provided on the conversion efficiency.

In general, compared to the actuation application, energy harvesting poses further requirements in terms of the material properties, such as the need for a very low electrical conductivity of the DE material, which has a tremendous impact on the generator efficiency.

In this communication we report on the fabrication process and performance testing of a class of DEGs made entirely of PDMS elastomers. The DEG topology under investigation is referred to as an inflated circular diaphragm DEG (ICD-DEG); i.e., a device able to harvest the mechanical work done by an oscillating pressure that inflates and deflates a circular membrane [24]. ICD-DEGs have previously been studied in laboratory tests [8,23] and in WEC applications in wave-tank basins [25] using natural rubber or acrylic as the DE material, while no evidence of their employment in combination with PDMS is present.

In the following, we describe a PDMS-based prototype of an ICD-DEG assembly built using commercial PDMS films and compounds. In particular, the active DE layer within the DEG is a commercial film (Elastosil^®^ 2030 by Wacker (München, Germany)) based on a two-part silicone formulation commonly used in DE actuator applications [20,26], while the compliant electrodes are manufactured using a carbon powder and PDMS blend laid on the DE layer in such a way as to form a compact multi-layer assembly. A simple and repeatable procedure for ICD-DEGs fabrication and assembly is first presented, followed by the experimental characterization of the developed prototype on a custom test-bench [27]. In the experiments, converted electrical energy densities over 150 J/kg were measured, with efficiencies up to 30%, thus hitting an unprecedented result in terms of PDMS-based DEGs performance.

The paper is organized as follows. Section 2 presents the materials and the manufacturing procedure for the composite PDMS system. Section 3 shows the experiments for its characterization. Section 4 describes and discusses the results of the experiments. Section 5 illustrates conclusions and future works.

## 2. Materials and Setup

For the manufacturing of the DEG, we employed a couple of 100 μm thick films of Elastosil^®^ 2030 commercialized by Wacker Polymers as dielectric layer, and we defined the following procedure to manufacture the ICD-DEG: (1) preparation of the materials for the electrodes; (2) deposition of the electrodes; (3) crosslinking in a vacuum oven; (4) assembly and mounting on a holding fixture.

**Figure 1:**
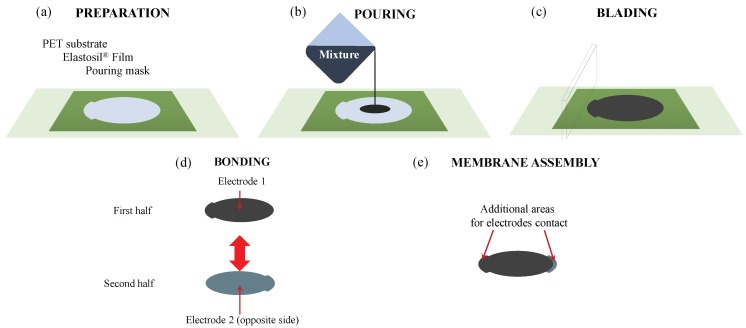
Phases of the preparation of the elastomeric electrode: (**a**) A pouring mask with a shaped cut is laid on the Elastosil^®^ film; (**b**) A mixture of polydimethylsiloxane (PDMS), carbon black, and isopropanol is poured; (**c**) The excess material is removed by blading; (**d**) After crosslinking, two membranes are bonded; (**e**) The membrane is assembled.

Deformable conductive electrodes are prepared with a formulation that is similar to the one proposed by Rosset et al. [19]. In detail, a mixture of carbon black powder (Cabot Vulcan XC72 (Cabot Corporation, Boston, MA, USA) ) and PDMS Wacker silicone Silgel^®^ RT625 (Wacker Chemie AG, München, Germany) was employed. Specifically, a liquid formulation was prepared using 0.8 g of an initiator, 8 g of polymer precursor, 32 g of isopropanol, and 0.8 g of carbon black. An amount of approximately 5 g of the prepared mixture was deposited on one of the surfaces of the DE film using a laser cut mask to shape the desired geometry of the electrode (i.e., a circle of 107 mm in diameter with an additional area for electrode connections). Excess material was removed through a blade, then the mask was taken off. The process (briefly illustrated in Figure 1) was identically repeated for two DE films. The two films holding the mixture were lightly degassed and cured for 12 h in an oven at 80 °C. When the process was completed, the obtained electrode featured an average thickness of 65 ± 25 μm and resistivity in the range of 10–20 kΩ/sq. The two films were finally overturned and oriented in such a way that the two electrode connections were arranged on opposite sides. The films were then jointed together on the electrode-free side to obtain a single 200 μm thick DE layer between the two deposited compliant electrodes. The natural self-adhesion of the silicone films is enough to keep them bonded. The overall thickness of the DEG assembly (DE layer and electrodes) was 330 μm.

**Figure 2:**
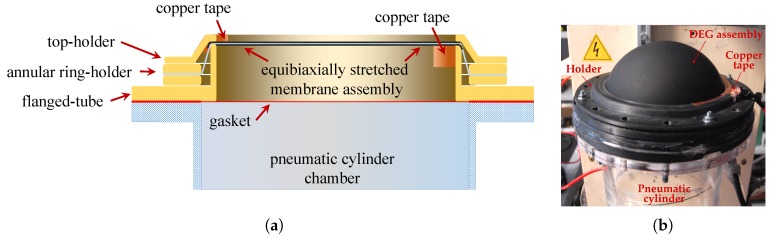
(**a**) Schematic of the membrane assembly (screws are not represented): the membrane assembly is secured on an annular ring holder and squeezed on a flanged-tube (that provides it with a certain pre-stretch), which is fixed to the ring-holder by means of a top holder. (**b**) A picture of the inflated dielectric elastomer generator (DEG) assembly during operation.

The membrane assembly (see Figure 2a) was mounted on a holder made of two annular rings that are bolted in such a way as to squeeze the external boundary of the membrane. A flanged tube with an internal diameter of 123 mm was then inserted and screwed onto the ring-holder to produce an out-of-plane deformation of the membrane and induce an equibiaxial pre-stretch of $\lambda_{p}=1.15$ in its central part. Finally, after pre-stretching, a further ring-shaped holder was bolted on the top in order to avoid slipping at the membrane perimeter. The contact with the electrodes is ensured by copper tape strips that are attached on the top of the flanged-tubes (high-voltage electrode) and on the external ring-shaped holder. An image of the deformed DEG assembly and of its holding structure is shown in Figure 2b.

**Figure 3:**
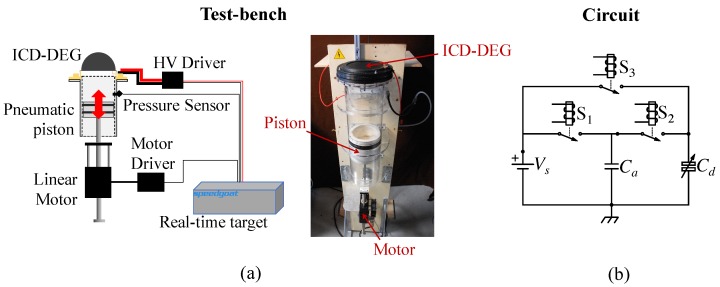
(**a**) Schematic and photograph of the experimental setup. (**b**) Schematic of the control circuit. In the picture, $V_{s}$ is the supply voltage, $C_{d}$ is the variable DEG capacitance, $C_{a}$ is the in-parallel capacitance, and S${}_{1}$, S${}_{2}$, S${}_{3}$ denote the switches. HV: high-voltage; ICD-DEG:inflated circular diaphragm DEG.

A DEG assembly which was prepared with the described procedure was fixed on a test-bench designed for the testing and characterization of ICD-DEGs [27] (see the scheme in Figure 3). This setup comprises: A mechanical sub-system that makes it possible to inflate and deflate the DEG membrane; a high-voltage (HV) electronics combined with a real-time controller that can drive the DEG and the mechanical system while synchronously acquiring the sensor signals.

The main component of the mechanical sub-system is a pneumatic actuator made of an air-tight movable piston with a diameter *D* = 130 mm, which slides into a polycarbonate cylinder tube with a flange at the top that hosts the DEG assembly. The pneumatic system is actuated via a brushless linear motor (P01-37x120F/200x280-HP by LinMot (Spreitenbach, Switzerland) ), with an embedded encoder that is used to measure the piston position. A pressure sensor (MPX12 by Freescale Semiconductor (Austin, TX, USA) ) installed on the cylinder tube is used to measure the differential pressure, *p*, between the cylinder chamber and ambient air. The pneumatic cylinder is mounted vertically and an elastic spring is introduced in order to compensate for the weight of both the piston and the motor slider. A schematic and a photograph of the mechanical sub-system are shown in Figure 3a.

The electrical regulation of the DEG is implemented through a HV power and sensing electronics whose schematic is represented in Figure 3b. The considered driving electronics comprises a four-quadrant high-speed HV power amplifier (Trek 10/10B-HS (TREK Inc., Lockport, NY, USA)) and a HV reed relays box (equipped with three HM12-1A69-150 by MEDER Electronics (Salem, NH, USA)) that alternatively connect the DEG electrodes to either the power supply or to an additional capacitor with capacitance $C_{a}=23.5\;\mathrm{nF}$ (whose role is clarified in the following). To measure the electric potential difference, $V_{d}$, across the DEG capacitance, $C_{d}$, a custom-made HV probe was implemented that features very high input resistance (nearly 50 GΩ), which limits the drain of charge from the ICD-DEG electrodes, and large bandwidth, which is obtained thanks to a tuned capacitor compensation network. Voltage sensing could be alternatively implemented using a capacitive sensor with very small capacitance. Potential advantages of capacitive sensing would be the absence of a draining current through the sensor, fast response time, immunity to electromagnetic interference, and high temperature stability. High-resolution capacitive sensing has been demonstrated in the literature using the differential capacitive sensor and switching sensor methods [28].

In order to minimize the current leakage of the power electronics, all the HV wirings in the presented setup and components have been encapsulated via thick layers of silicone gel (Magic-gel by Raytech (Milan, Italy)) and acrylic tape (VHB 4905 by 3M). A real-time machine by SpeedGoat (Liebefeld, Switzerland) ^®^ running the MatLab ^®^ 2014b xPC Target software environment was employed to control the motion of the piston, the HV power supply, and the relays.

## 3. Experimental

**Figure 4:**
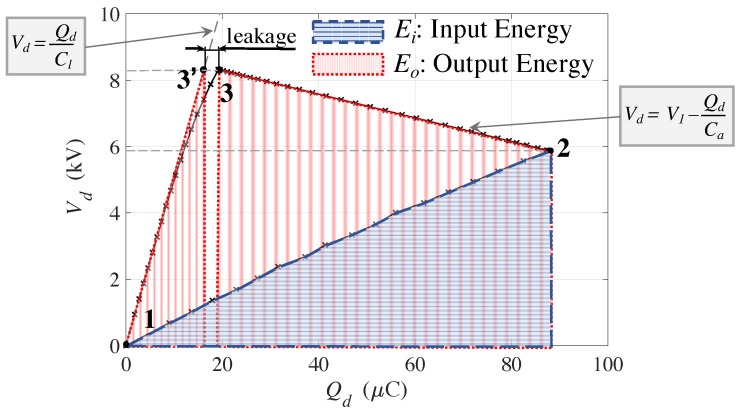
Charge voltage Q−V plot of a sample generation cycle. It is worth noting that only the states **2** and **3’** are actual physical states of the DEG, while **3** is a point such that $V_{3}=V_{3^{\prime }}$ and $Q_{3}=Q_{2}-C_{a}\left(V_{3}-V_{2}\right)$. The points **3** and **3’** coincide in the case of the absence of charge leaks during the generation phase.

The generation cycle is implemented as shown in Figure 4. The switches $S_{1}$ and $S_{2}$ are opened, $S_{3}$ is closed, and the voltage of the supply is set to $V_{s}=0$ (i.e., the DEG voltage is also $V_{d}=0$). The piston is moved upward at constant velocity by a quantity *z*, and consequently the DEG membrane is inflated (i.e., the capacitance is increased to a value $C_{h}$). The switch $S_{3}$ is opened (i.e., the DEG is isolated and $S_{1}$ is closed and the capacitor $C_{a}$ is charged), with a supply voltage $V_{s}=V_{1}$ (during this operation the piston maintains its position). The capacitor $C_{a}$ is disconnected from the power supply by opening $S_{1}$ and connected to the DEG by closing $S_{2}$, thus the DEG is rapidly charged (1→2) up to a voltage $V_{2}$. Then, the the piston returns to its home position and the DEG (in parallel with $C_{a}$) increases its voltage to $V_{3}$ and reaches the flat configuration ($2\to 3/3^{\prime }$), corresponding to the state where the DEG exhibits minimum capacitance $C_{l}$. Lastly, the DEG is isolated by opening $S_{2}$, the supply voltage is set to $V_{s}=0$, and the DEG is discharged by closing $S_{3}$, draining charge through the (bidirectional) power supply ($3^{\prime }\to 1$). It is worth noting that the additional capacitor $C_{a}$ assumes two different roles in the generation cycle. First, it is employed in the priming of the DEG, making it possible to estimate the amount of charge that is transferred. Second, it is employed during the generation phase to impose a linear trajectory on the Q−V plane (2–3) that makes it possible to increase the amount of harvestable energy [9]. The whole cycle lasts 2 s, and is repeated four times.

**Figure 5:**
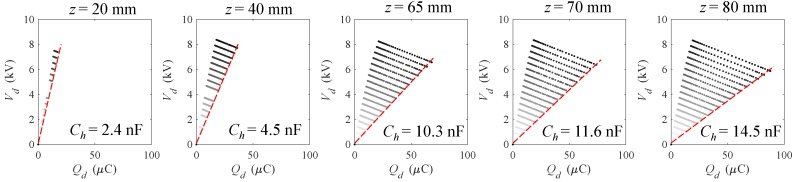
Generation cycles on the Q−V plane and indication of the evaluated capacitance $C_{h}$ of the inflated DEG during the priming phase. Note that the value of the DEG capacitance in the flat configuration was measured with the same procedure at z=0 and resulted in $C_{l}=1.9\;\mathrm{nF}$. Some tests at intermediate voltages are missing for piston displacements of 20 mm and 40 mm due to a few faults in the data acquisition.

Experiments have been conducted for increasing values of the supply voltage, $V_{1}$, and of the piston displacement, *z*, respectively, in the ranges of 1–9 kV (with increments of 0.5 kV) and 0–80 mm (with uneven increments). These correspond to a maximum electric field, calculated when the membrane is in the flat condition, of 54.6 MV/m which is a relatively small value compared to the breakdown field of 80–100 MV/m that is declared in the product data-sheet by the manufacturer. This moderate electrical loading is likely to guarantee a long lifetime of the system—on the order of millions of cycles [29]. The measured generation cycles are represented on the Q−V plane in Figure 5, where the different plots refer to different inflation levels, whereas the different lines in the same plot refer to different priming voltages.

## 4. Results and Discussion

**Figure 6:**
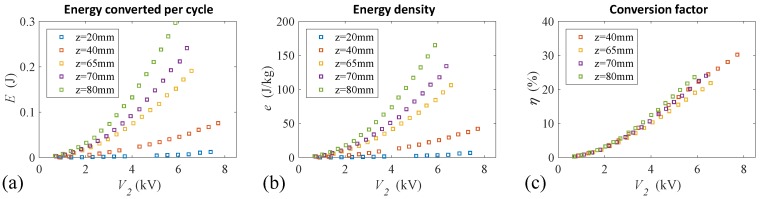
Figures of merit for different inflation levels (calculated as the average over four cycles) plotted against the priming voltage: (**a**) Converted energy; (**b**) Energy density; (**c**) Conversion factor (values for z=20mm are not reported since the relative small amount of the energy generated makes the estimation unreliable in this case).

In order to evaluate the generation performance of the developed system, relevant figures of merit have been calculated through an elaboration of the experimental results according to the following procedures. The electrical energy that is converted in each generation cycle is calculated as the difference between the output energy, $E_{o}$, and the priming energy, $E_{i}$:

(1) $$E\approx \underset{E_{o}}{\underset{︸}{\frac{1}{2}C_{a}\left(V_{3}^{2}-V_{2}^{2}\right)+\frac{1}{2}C_{l}V_{3}^{2}}}-\underset{E_{i}}{\underset{︸}{\frac{1}{2}C_{h}V_{2}^{2}}}$$

where $C_{l}$ and $C_{h}$ are the values of the DEG capacitance in the flat and fully inflated conditions. It is interesting to underline that this estimation is not accounting for the effect of the membrane expansion due to the supplied electrical activation [30], because this is negligible in these experimental conditions. The mass energy density is calculated as $e=E/m_{d}$, with $m_{d}=1.7\;g$ being the active mass of DE material that is employed; the conversion factor is η=E/W, where *W* is the mechanical work input that is done by the pneumatic piston over a cycle, estimated from the time series of piston position and cylinder chamber pressure. The values of the figures of merit obtained from the experimental tests are reported in Figure 6 against the initial priming voltage, with different lines corresponding to different levels of inflation. Overall, a maximum value of 0.3 J for the converted energy per cycle (which corresponds to 0.15 W of average power since the frequency of the cycles is 0.5 Hz) has been obtained for a capacitance variation by a factor 7.6. A maximum value of 173 J/kg was measured for the energy density. This is an extremely high value compared to existing results reported to-date for PDMS-based DEGs [7,21] (e.g., in [7] an energy density of 3.5 J/kg and a power density of roughly 3.8 W/g are claimed), and they are on the same order of magnitude as the maximum energy densities measured with acrylic and natural rubber elastomers, for which values of up to 780 [9] and 370 J/kg [8] have been measured, using larger strains and electric fields closer to the break-down condition than those employed in the present experiments. The considered figures of merit are monotonically increasing with the voltage, according to a typical quadratic-shaped response, and with the level of inflation. As for the conversion factors, one can notice that values as high as 30% are reached for the smaller inflation levels. This high value is obtained thanks to the control [9] of charge and voltage achieved by introducing the additional in-parallel capacitor, $C_{a}$, in the generation circuit. The lost energy is mainly attributed to mechanical hysteresis and air-leaks, with this second effect having a relevant share. As shown in Figure 4, electrical inefficiencies are nearly negligible, since charge leakages are relatively small, with a maximum of 3μC (corresponding to 25 mJ) of the priming charge. In future experiments, the efficiency could be largely increased by improving the air-tightness.

## 5. Conclusions

This communication presents the fabrication procedure and experimental performance characterization of a dielectric elastomer generator (DEG) made of polydimethylsiloxane (PDMS) compounds. The DEG assembly is a composite membrane that integrates a commercial dielectric elastomer film (namely, Elastosil^®^ 2030) and a couple of compliant electrodes obtained by mixing a commercial two-part PDMS blend (Silgel^®^ RT625) with carbon powder. Mechanically, the DEG is an inflating membrane, referred to as inflated circular diaphragm DEG (ICD-DEG). The commercial Elastosil^®^ 2030 film is purposely commercialized by Wacker for DE applications, and to-date it has been investigated for sensing and actuation applications only. The present work represents the first example of its usage for generation purposes.

The communication presents a set of generation experiments performed on the ICD-DEG sample using a custom-made test-bench, and it shows some relevant figures of merit for the device performance (namely, the cyclic converted energy and energy density, and the electro-mechanical conversion factor). The prototype has delivered a convertible energy density of up to 173 J/kg, which is several tens of times larger than that previously reached with PDMS-based DEGs, and a mechanical-to-electrical energy conversion factor of up to 30%. The measured energy density is on the same order of magnitude of that previously measured on DEG prototypes based on acrylic or natural rubber DEs, even though conservative levels of electrical loading of the PDMS dielectric material (compared to the break-down material limits) have been employed in the reported tests.

Future work will focus on the design and manufacturing of an upscaled prototype/set-up of the system, and the characterization of the long-term stability and fatigue lifetime of the DEG assembly.

## Abbreviations

The following abbreviations are used in this manuscript:

DE	Dielectric Elastomer
DEG	Dielectric Elasotomer Generator
ICD-DEG	Inflatable Circular Diaphragm Dielectric Elasotomer Generator
PDMS	Polydimethylsiloxane
